# Impact of a non-contributory pension for older adults on hospitalisations and mortality: a protocol for a natural experimental study of the Brazilian continuous cash benefit (BPC)

**DOI:** 10.1136/bmjopen-2026-119914

**Published:** 2026-07-03

**Authors:** Samuel Araujo Gomes da Silva, Francine de Souza Dias, Joanna M N Guimarães, Gustavo Casais, Ana L Moncayo, Ronal Alejandro Grijalva Ruiz, Daiane Borges Machado, Flávia Jôsi Oliveira Alves, Srinivasa Vittal Katikireddi, Alastair H Leyland, Maurício Lima Barreto, Rita de Cássia Ribeiro, Peter Craig, Julia Pescarini

**Affiliations:** 1 Centro de Integração de Dados e Conhecimentos para Saúde (Cidacs), FIOCRUZ Bahia, Salvador, Bahia, Brazil; 2 National School of Public Health, Oswaldo Cruz Foundation, Rio de Janeiro, Brazil; 3 Faculdade de Economia, Universidade Federal da Bahia, Salvador, Brazil; 4 Centro de Investigación para la Salud en América Latina (CISeAL), Pontificia Universidad Católica del Ecuador, Quito, Ecuador; 5 Universidad Internacional del Ecuador UIDE, Quito, Ecuador; 6 Department of Global Health and Social Medicine, Harvard Medical School, Boston, Massachusetts, USA; 7 MRC/CSO Social & Public Health Sciences Unit, University of Glasgow, Glasgow, Scotland, UK; 8 Universidade Federal da Bahia, Salvador, Brazil; 9 MRC/CO Social and Public Health Sciences Unit, Glasgow University, Glasgow, UK; 10 Department of Infectious Disease Epidemiology & International Health, London School of Hygiene & Tropical Medicine, London, UK

**Keywords:** Aged, Health Impact Assessment, Social Support

## Abstract

**Abstract:**

**Introduction:**

The continuous cash benefit (Benefício de Prestação Continuada, BPC) is Brazil’s main non-contributory social pension. It guarantees a monthly income equivalent to one minimum wage to individuals aged ≥65 years and to people with disabilities living in poverty, but evidence on its health effects remains limited. This protocol describes the design and methods for evaluating the effects of BPC on hospitalisations and mortality among older adults and their household members.

**Methods and analysis:**

This natural experiment study uses linked administrative records from the 100 Million Brazilian Cohort (2001–2021), BPC payments (2016–2021), mortality (2001–2020) and hospitalisation data (2008–2018). Eligibility for elders is defined by age (≥65 years) and monthly family per capita income (<¼ of the minimum wage or up to 1/2 the minimum wage for specified groups). We defined as treated individuals who initiate BPC receipt from 2016 onwards. Untreated individuals are income or age-eligible or near-eligible adults who do not receive the benefit. The study population comprised people who were registered at any time in the cohort but were still alive on 1 January 2016. We triangulate two approaches. First, we will employ fuzzy regression discontinuity design using age and income eligibility thresholds to estimate local average treatment effects for different bandwidths. To assess the continuity assumption, we will analyse individuals aged 60 years or older to test outcome smoothness, while eligibility remains defined at age 65. Second, we will employ survival models with inverse probability of treatment weighting to estimate effects under conditional exchangeability assumptions. Outcomes include all-cause and cause-specific mortality and hospitalisations among older adults and their co-inhabitants. Analyses account for household clustering using cluster-robust SEs. Sensitivity analyses will assess robustness to data quality, municipal characteristics and potential overlap with Bolsa Família receipt.

**Ethics and dissemination:**

This study was approved by the Brazilian National Research Ethics Commission (CONEP) (approval number: 85981225.6.0000.0040), in accordance with the Brazilian National Health Council Resolution No. 466/2012. Findings will be disseminated through peer-reviewed publications, conference presentations, and policy briefs targeting stakeholders in social protection and health in Brazil. Results will also be shared with relevant governmental and non-governmental actors to support evidence-informed decision-making.

**STRENGTHS AND LIMITATIONS OF THIS STUDY**

- This study leverages large-scale linked administrative data from the 100 Million Brazilian Cohort, enabling high statistical power and detailed population coverage.
- The use of a fuzzy regression discontinuity design (RDD) exploiting age and income eligibility thresholds strengthens causal inference by addressing both observed and unobserved confounding near the cut-off.
- Triangulation with propensity score-based methods (inverse probability of treatment weighting) allows assessment of robustness under different identification assumptions and improves external validity.
- The RDD approach estimates local average treatment effects near eligibility thresholds, which may limit generalisability to the broader population of beneficiaries.
- Administrative data limitations, including potential measurement error in income and incomplete capture of hospitalisations outside the public health system, may affect outcome ascertainment, although this limitation is likely to be limited given that the study population relies predominantly on Brazil’s public health system (Sistema Único de Saúde).

## Introduction

 Like many other low- and middle-income countries (LMICs), Brazil is undergoing significant demographic changes, marked by an increasing proportion of older individuals,^1^ with individuals aged 65 years and older accounting for 10.9% of the national population in 2022, a 57.4% increase compared with 2010.^2^ Beyond the biological processes of ageing, poorer health outcomes observed among specific subgroups of older adults are largely driven by structural and socioeconomic inequalities, including barriers to healthcare, education, adequate food and housing, social assistance, transportation and access to information.^3–5^ These factors reflect cumulative disadvantage across the life course rather than ageing per se, highlighting the heterogeneity of health trajectories in later life.

Social protection policies play a crucial role in addressing socioeconomic disadvantages faced by older individuals. Internationally, social protection systems are recognised as key instruments for ensuring income security, dignity and autonomy, particularly for those who experience functional limitations.^6^ Over the past decades, social protection has expanded significantly across the Global South, reshaping welfare debates beyond the traditional European model, whose institutional logic has proven difficult to replicate in distinct economic, political and historical contexts.^7^ Throughout much of the 20th century, protection systems in many LMICs remained fragmented and limited to formal workers, excluding large segments of the population.^8^ Since the late 1990s, however, the expansion of social assistance programmes, particularly cash transfers and unremarkable pensions, has reshaped welfare landscapes across middle-income countries such as Brazil, China, India and South Africa.^7 9^

In Latin America, both conditional and unconditional cash transfer programmes have been central to poverty reduction and social inclusion strategies.^10^ Conditional cash transfers (CCTs), which typically target low-income families with children and include co-responsibilities in health and education, have been particularly prominent. Brazil’s Bolsa Família Programme (BFP) stands out as a widely studied example.^11 12^ While BFP is a CCT targeting low-income families with children, Brazil’s Continuous Cash Benefit (Benefício de Prestação Continuada, BPC) operates as a unremarkable, unconditional social assistance benefit guaranteeing a minimum income to older individuals and people with disabilities living in poverty.^12^

Unlike conditional transfers, the BPC is established as an individual right, addressing both income insecurity and disability-related costs.^6^ The programme is central to Brazil’s welfare architecture due to its potential to mitigate vulnerabilities such as healthcare expenses, long-term care and social participation barriers. However, empirical evidence on the programme’s broader impacts remains scarce. A recent scoping review including studies evaluating implementation or effects of BPC on health and social determinants of health identified only a small body of evidence.^12^ Evidence from 13 studies suggests BPC benefits on poverty reduction, income inequality and household well-being, but the use of rigorous causal inference methods was scarce^13–15^ and there was no individual-level study evaluation on the effects of the programme on mortality or hospitalisations.^12^ This underscores the need for more rigorous evaluations of planned and unplanned impacts within Brazil’s social protection system.^12^

This protocol proposes a comprehensive methodological framework to estimate the causal effects of BPC on all-cause hospitalisations and mortality among older adults across Brazil, as well as to examine the most common causes of morbidity and mortality in this group.^16^ Advancing beyond prior evaluations, this study leverages the 100 Million Brazilian Cohort and uses a natural experiment aiming to estimate the causal effect of the BPC on health, mitigating the effects of unmeasured confounding. In doing so, the study aims to inform policy debates, strengthen social welfare strategies and advance health equity for structurally vulnerable populations.

## Methods

This protocol follows the TIDieR-PHP (Template for Intervention Description and Replication for Population Health and Policy Interventions) guideline,^17^ ensuring a comprehensive and replicable description of the intervention and methods. The study employs a quasi-experimental design and leverages data from multiple large-scale administrative databases to evaluate the impact of the BPC on health outcomes, including hospitalisations and mortality among beneficiaries and their household members.

### Intervention

BPC is a unremarkable social pension established under Brazil’s *Lei Orgânica da Assistência Social* in 1993, with implementation beginning in 1996.^18^ It is granted within the framework of Brazil’s social assistance policy and operationalised through agencies of the National Institute of Social Security (INSS), which also administers contributory social insurance programmes. BPC guarantees a monthly income equivalent to one Brazilian minimum wage (BRL1621.00, approximately US$315 in 2026) to individuals living in extreme poverty who meet one of the following criteria: (1) being aged 65 years or older, regardless of prior participation in contributory social insurance or pension schemes or (2) having a disability that results in long-term impairments (lasting at least 2 years) that limit full and effective participation in society on equal terms with others.

In addition to their age and disability status, eligibility criteria for both groups depend on meeting the stipulated family income threshold, typically a monthly per capita household income of up to 1/4 of the minimum wage (BRL405 or ~US$78 in 2026). However, for individuals meeting specific conditions (Brasil, 2021), this threshold can be increased to 1/2 of the minimum wage (BRL810 or ~US$157 in 2026). Such conditions include the severity of the disability, dependence on third-party assistance for daily activities and significant household expenditures on medical treatments.^19 20^ This flexibility in the income criterion seeks to address heightened social vulnerability and ensure broader access to the benefits. However, it has reshaped the profile of beneficiaries and affected comparability across the cohort over time. Finally, since 2010, BPC beneficiaries are also eligible for 10%–65% discounts on electricity tariffs.^21^

It is essential to highlight that, within the BPC, the family unit is formally defined as individuals residing in the same household and sharing income and expenses, including the applicant, their spouse or partner, parents, siblings, children and grandchildren. The per capita family income is calculated by summing the total monthly income of the family unit and dividing it by the number of individuals in the household. In 2016, registration in the Unified Registry for Social Programmes (Cadastro Único, CadÚnico) became mandatory to access the benefit.^22 23^ (See online supplemental table S1 for a full description of BPC and its variations in the TIDieR-PHP template).

### Conceptual framework

Although the programme’s legal framework does not explicitly detail the mechanisms by which the BPC may influence health, the benefit forms part of a broader social assistance strategy aimed at improving population well-being.^24^ In this study, the programme framework reflects our analytical understanding of how the BPC operates and how its components may contribute to changes in beneficiaries’ living conditions. Specifically, we consider programme inputs (public financing via the National Social Assistance Fund, administrative capacity of Reference Centres for Social Assistance (CRAS) and INSS and the medical-social assessments conducted by the INSS), operational mechanisms (ie, identifying eligible individuals, verifying income and disability status and delivering monthly cash transfers); intermediate living conditions; and resulting health outcomes for beneficiaries and their households. Immediate outputs, such as the timely inclusion of eligible low-income older adults and the delivery of monthly cash transfers, may vary according to area-level and municipality-level deprivation, which may reflect differences in local managerial capacity.^25^ These outputs are expected to translate into intermediate outcomes, including the alleviation of household poverty and reductions in income instability.

The reduction of financial deprivation initiates a set of intermediate improvements in living conditions that are key social determinants of health. We hypothesise that this includes improvements in access to food, housing, sanitation and healthcare services, as well as reduced financial stress and greater social participation. Conceptual frameworks on social protection and health emphasise that income security across the life course, particularly in older age, shapes both material conditions and psychosocial pathways that are central to healthy ageing and health equity.^26^ In Latin America, analyses of social protection systems highlight the role of unremarkable pensions in reducing poverty and improving quality of life among older adults, often operating in interaction with health services, family arrangements and care systems.^27 28^ Empirical evidence on Brazil’s BPC, although still limited, also suggests positive effects on food security, reductions in income inequality, decreases in labour force participation of older adults, increased school participation among household members and improvements in health indicators and healthy life expectancy among older adults.^12^ In addition, a qualitative study focused on people with disabilities documented perceived gains in autonomy, dignity and social participation associated with the BPC, offering in-depth insights into psychosocial and material mechanisms through which income security may operate.^29^

**Figure 1:**
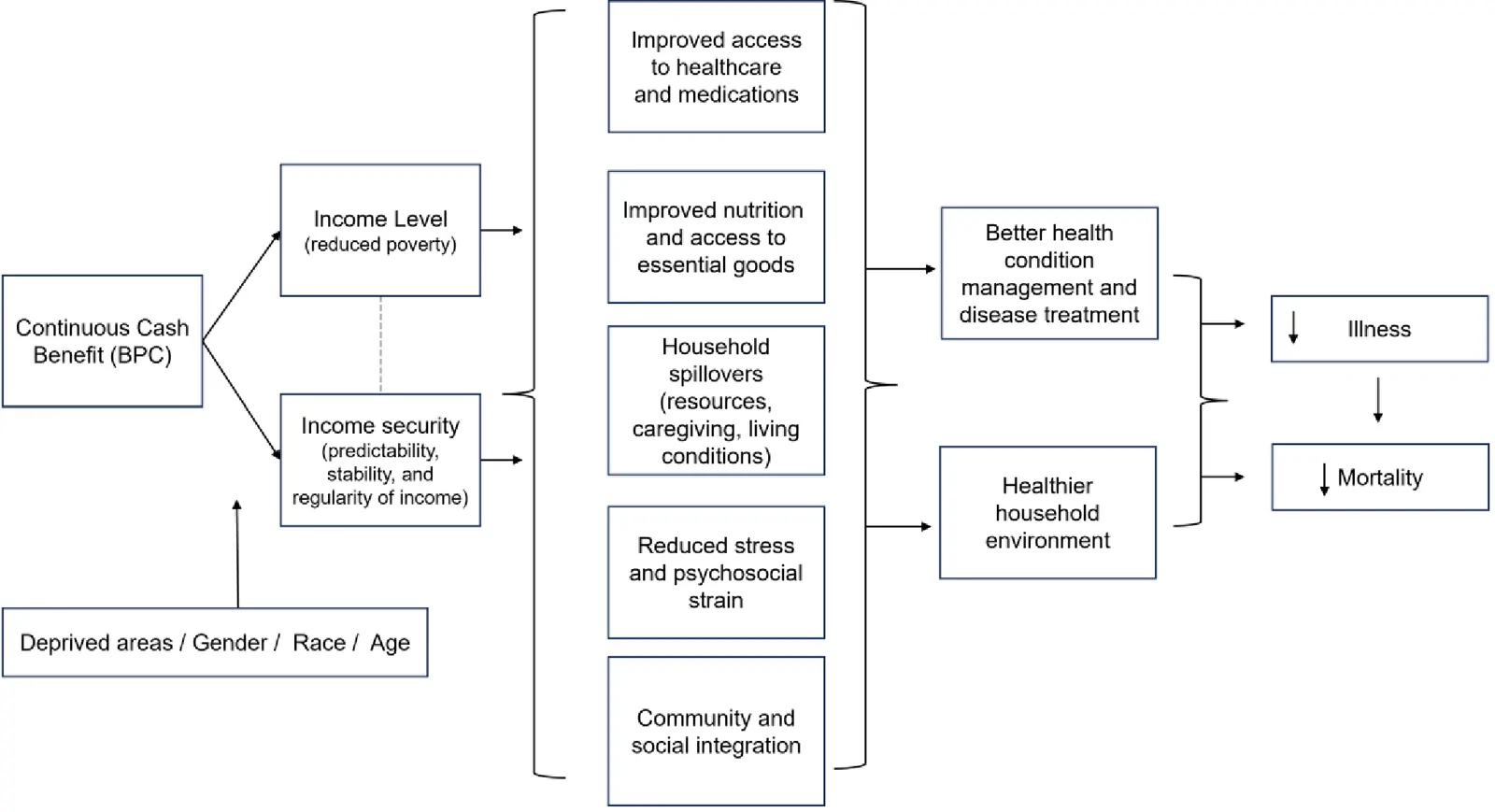
Logic model for the effects of the continuous cash benefit (BPC) on hospitalisation and mortality of older people and their household members. BPC, Benefício de Prestação Continuada.

These pathways have the potential to improve health, including reductions in premature mortality and changes in hospitalisation rates among beneficiaries. The mechanisms linking BPC to mortality (figure 1) are expected to operate primarily through improved material living conditions, increased food security, reduced stress associated with financial instability and enhanced capacity to afford transportation, medications and basic healthcare needs. In contrast, hospitalisation is a more complex outcome, as it may reflect changes in access to healthcare or in underlying health conditions. On the one hand, income stabilisation may reduce preventable hospitalisations by improving nutrition, treatment adherence and disease management. On the other hand, greater financial security may increase healthcare-seeking behaviour and facilitate access to hospital services, potentially leading to higher short-term admission rates. In the Brazilian context, where this population relies predominantly on the public health system (Sistema Único de Saúde, SUS), access is less directly constrained by healthcare costs and more by non-financial barriers, such as transportation, information and the ability to navigate services. We therefore interpret changes in hospitalisation rates cautiously, considering both reductions in avoidable admissions and potential increases related to improved access to care.

In multigenerational households, income received from BPC by older adults may be shared to support other co-resident members, which could attenuate the direct health benefits for the older recipient while generating broader household-level effects through spillovers to co-resident family members, particularly children, from improved food consumption, higher school attendance and reduced child labour. Evidence shows improvements in schooling outcomes and reductions in child labour among households where older adults receive unremarkable pensions in Brazil.^30 31^ Although empirical evidence on the health impacts of BPC remains limited, existing studies suggest meaningful improvements in poverty, food security and household well-being,^9 32^ and increases in healthy life expectancy among older adults.^13^ Therefore, we hypothesise that BPC contributes not only to reducing poverty and inequality but also to improving general well-being and quality of life, and ultimately to reducing morbidity and mortality among both beneficiaries and co-residents.

### Ethics and dissemination

This study was approved by the Brazilian National Research Ethics Commission (CONEP) (approval number: 85981225.6.0000.0040), in accordance with the Brazilian National Health Council Resolution No. 466/2012. Results will be disseminated through publication in peer-reviewed journals, presentations at national and international conferences and the production of policy briefs. These briefs will be specifically tailored for managers in the Brazilian Ministry of Health and Ministry of Social Development to support evidence-informed decision-making in social protection programmes.

### Patient and public involvement

The NIHR Global Health Research Unit on Social and Environmental Determinants of Health Inequalities has established a Community Engagement Advisory Committee. This group consists of approximately 25 stakeholders, including community leaders from different regions of Brazil, representatives of intergovernmental organisations, health professionals, managers and researchers. The research questions and the general protocol for this study were presented to and discussed with this committee during bimonthly online meetings and annual in-person workshops to ensure that the research agenda remains aligned with the needs of the affected communities and to prioritise policy evaluations. Since this specific study relies exclusively on the analysis of unidentified secondary administrative data, patients whose records are in the database were not directly involved in the design or conduct of this phase. There are no plans to disseminate results directly to individual study participants due to the scale and nature of the data; however, all findings will be discussed and validated with the Community Engagement Advisory Committee to co-produce tailored dissemination products, such as policy briefs, aimed at influencing regional and national social protection policies

### Study design and datasets

The study uses, for the first time, a dataset created by linking the BPC payment records to the large-scale population-based 100 Million Brazilian Cohort, hosted at the Centre for Data and Knowledge Integration for Health, Oswaldo Cruz Foundation (Fiocruz).

The 100 Million Brazilian Cohort baseline is a comprehensive dataset obtained from CadÚnico, encompassing over 140 million economically vulnerable individuals (2001–2021).^33^ The CadÚnico identifies low-income households, and registration can be completed at the CRAS or through home visits conducted by social workers. Households with per capita income up to half the minimum wage (R$760 or ~US$141 in 2025) or total family income up to three minimum wages (R$4554 or ~US$840 in 2025) are eligible to be registered with CadÚnico. Additionally, people experiencing homelessness, indigenous populations, and ‘quilombola’ communities might be registered even if their income exceeds these thresholds. Individuals must update their information if changes in household composition or income occur, or at a maximum of 2 years, to avoid benefit suspension. CadÚnico is an extensive database that includes housing, income, employment, education and public service access information. Therefore, the cohort baseline data provide information on demographic, socioeconomic and housing conditions, offering a rich context for studying social determinants of health.

In addition to cohort baseline data and its updates, the study links information from the Mortality Information System (SIM) (2001 to 2020) and the Hospitalisation System (SIH) (2008 to 2018). Given the available follow-up period (maximum of 4 years for some individuals and often shorter), analyses will primarily capture short-term effects of BPC receipt, with limited ability to assess longer-term impacts. The SIM provides detailed mortality data, including causes of death coded according to the 10th Edition of the International Classification of Diseases (ICD-10) standards^34^ and demographic information, with a high completeness rate (≥90%) across most municipalities (93%), despite regional variability.^35 36^ The SIH dataset contains hospitalisation records from Brazil’s public health system (SUS), including diagnoses, procedures and lengths of stay. Given that the 100 Million Brazilian Cohort consists predominantly of low-income individuals who rely primarily on the SUS for healthcare,^33^ particularly for hospital and emergency services, the SIH provides a valuable source of information for analysing patterns of healthcare utilisation^37^ among BPC beneficiaries.

From the BPC payment database, obtained through an agreement with Brazil’s Ministry of Social Development, we will extract annual information on the value of the benefit transfers starting from 1 December 2016, which is the earliest date BPC data was available with detailed linkable information. The dataset also includes sociodemographic characteristics such as age and sex along with details from the application process, including number of dependents, type of benefit (elderly or people with disabilities), and key dates such as application, approval, first payment and, in some cases, cessation (see table 1 for variables of the BPC database). Linkage between the cohort and the BPC data (restricted to the older people benefit) was performed using exact matching on the National Identification Number (NIS) available in the Cadúnico and social benefits databases. After linkage, 3 266 555 older individuals were successfully matched to the 100 Million Brazilian Cohort, which comprises 18 495 520 individuals in the corresponding age range. A total of 540 646 records (14.2%) from the BPC payment database remained unmatched due to missing or inconsistent identifiers. As linkage was based on exact linkage using the NIS, unlinked records likely reflect data quality issues rather than systematic exclusion. Sensitivity analyses will be conducted to assess potential bias arising from unlinked records, including comparisons of available characteristics between linked and unlinked individuals. Among the 3 266 555 successfully linked older individuals, 804 617 initiated BPC receipt between 2016 and 2020; this subgroup defines the treated group used in subsequent analyses.

**Table 1:** Variables contained in the BPC database

Variable description	Type
National Identification Number	Unique identifier (string)
Birth date	DD/MM/YYYY
Benefit value	Numeric
Benefit transferred	Yes/no
Age	Numeric
Sex	1–Male 2–Female
Date of application submission	DD/MM/YYYY
Benefit approval date	DD/MM/YYYY
Benefit start date	DD/MM/YYYY
Municipality of residence	Code (string)
State of residence	Code (string) for the 27 Brazilian states
Number of dependents	Number
Type of benefit granting	1–Social assistance benefit for persons with disabilities 2–Social assistance benefit for older adults

BPC, Benefício de Prestação Continuada.

### Study population

The study population includes individuals registered in the 100 Million Brazilian Cohort and in the BPC database. The follow-up period for this study will start on 1 January 2016, aligning with the mandatory registration of BPC applicants in the CadÚnico. The primary analysis will include individuals entering the cohort until 31 December 2020, with outcome monitoring (mortality and hospitalisations) extending to the latest available data in the SIM (currently 31 December 2020) and SIH systems (currently 31 December 2018) and treatment defined as initiation of BPC receipt from 2016 onwards.

Exclusion criteria are applied to address potential data inconsistencies. These criteria include:

1. individuals who died before registering with the cohort, as anomalous dates could reflect linkage errors;
2. those aged 100 years or older at registration, most likely due to missing or mismatched death certificates;
3. individuals who registered on the last follow-up day or died on the same day they registered;
4. individuals who initiated BPC receipt before 2016, to avoid including individuals with prior treatment.

Follow-up will end when individuals reach 100 years of age, considering the rarity (0.018%) of this age group in Brazil^38^ and the potential for linkage errors beyond this point.^39^

Therefore, our analytical cohort comprises individuals who registered with the 100 Million Brazilian cohort and survived until 1 January 2016. Treatment was then defined as whether either they or an older person in their household started receiving BPC from that date onwards (see table 2 for the description of age, sex distribution, and income percentiles of the analytical cohort). Importantly, since 2011, improvements in CadÚnico’s data collection procedures, income verification routines and periodic updating requirements have strengthened the quality and reliability of reported income information, increasing confidence in its use for eligibility-based analyses.

**Table 2:** Demographic and income distribution of the people aged 60–100 years in the 100 million cohort (100M Cohort) and BPC beneficiary samples, including the 2016–2021 analytical window

	People aged 60–100 in the 100M Cohort (2001–2020) (n=18 495 520)		People aged 60–100 in the 100M Cohort (2016–2020) (n=3 975 617)		Beneficiaries of BPC for older people (linked to the 100M Cohort) (n=3 266 555)		Analytical cohort: beneficiaries of BPC for older people starting to receive benefits between 2016 and 2020 (linked to the 100M Cohort*) (n=804 617)	
Age group	n	%	n	%	n	%	n	%
60–64	5.234.516	28.30	998.239	25.11	576.457	17.65	382	0.05
65–69	4.345.339	23.49	1.026.468	25.82	843.402	25.82	625.016	77.69
70–74	3.318.755	17.94	758.923	19.09	705.099	21.59	147.925	18.39
75–79	2.284.352	12.35	522.762	13.15	536.159	16.42	21.948	2.73
80–84	1.575.944	8.52	362.652	9.12	358.320	10.97	6.474	0.80
85–89	888.435	4.80	188.479	4.74	173.162	5.30	2.073	0.26
90+	848.179	4.59	118.094	2.97	73.675	2.26	705	0.09
Sex								
Male	8.489.780	45.9	1.874.544	47.15	1.332.075	40.78	342.797	42.60
Female	10.005.740	54.1	2.101.073	52.85	1.934.451	59.22	461.819	57.40
Baseline income prior to BPC (R$). Standardised in minimum wages by year of entry								
1%	0	*	0	*	0	*	0	*
5%	0	5	0	5	0	5	0	5
10%	0	10	0.68	10	0	10	0	10
25%	0.08	25	0.39	25	0.32	25	0.07	25
50%	0.75	50	0.85	50	*	50	0.35	50
75%	12.85	75	*	75	9.65	75	1.02	75
90%	46.45	90	1.17	90	87.62	90	40.19	90
95%	76.36	95	1.57	95	100	95	55.78	95
99%	126.105	99	2.55	99	110.61	99	102.75	99

* Individuals who entered the cohort baseline and had their BPC applications approved between 2016 and 2021.

BPC, Benefício de Prestação Continuada.

#### Primary and secondary outcomes

The two primary outcomes are (1) all-cause hospitalisations and (2) all-cause mortality. Secondary outcomes include cause-specific mortality or cause-specific hospitalisations, classified using the ICD-10^34^ (see table 3 for ICD-10 codes included in this analysis). As individuals can have multiple diagnostic codes for a hospitalisation, to address concerns about coding quality, we will focus on the primary diagnosis codes for hospitalisations, although sensitivity analyses including secondary diagnoses will also be conducted. Individuals may experience more than one hospitalisation during the follow-up period; therefore, multiple events will be considered in the analysis rather than restricting it to the first occurrence only. To disentangle whether changes in hospitalisation rates reflect improved healthcare access or worsening health, we will categorise hospitalisations into preventable and non-preventable causes. A reduction in admissions for primary care-sensitive conditions will be interpreted as improved health management, while a potential short-term increase in non-preventable admissions may reflect enhanced access to hospital services enabled by increased household income.^40^

**Table 3:** Causes of hospitalisation and death that shall be investigated in this protocol according to ICD-10 codes

Causes of hospitalisation and death	ICD-10 codes
**1. All-cause mortality**	**All**
**2. Chapter I: Certain infectious and parasitic diseases**	**A00–B99**
**3. Chapter II: Neoplasms**	**C00–D48**
3.1 Malignant neoplasms	C00–C97
**4. Chapter IV: Endocrine, nutritional and metabolic diseases**	**E00–E90**
4.1 Diabetes mellitus	E08–E13
4.2 Malnutrition	E4–E46
**5. Chapter V: Mental and behavioural disorders**	**F00–F99**
**6. Chapter VI: Diseases of the nervous system**	**G00–G99**
**7. Chapter IX: Diseases of the circulatory system**	**I00–I99**
7.1 Hypertensive diseases	I10–I15
7.2 Ischaemic heart diseases	I20–I25
7.3 Cerebrovascular diseases	I60–I69
**8. Chapter X: Diseases of the respiratory system**	**J00–J99**
8.1 Influenza and pneumonia	J09–J18
**9. Chapter XI: Diseases of the digestive system**	**K00–K93**
9.1 Noninfective enteritis and colitis	K50–K52
**10. Chapter XIV: Diseases of the genitourinary system**	**N00–N99**
**11. Chapter XIX: Injury, poisoning and certain other consequences of external causes**	**S00–T98**
**12. Chapter XX: External causes of morbidity and mortality**	**V01–Y98**
12.1 Falls and accidents	V00–X59
12.2 Falls	W00–W19
12.3 Suicide	X60–X84
12.4 Homicide	X85–Y09

ICD-10, 10th Edition of the International Classification of Diseases.

For mortality, we will analyse the underlying cause of death (ICD-10) recorded on official death certificates to ensure consistency and accuracy in the attribution of outcomes. Through linkage with the SIM, all deaths among individuals who entered the analytic baseline between 1 January 2016 and 31 December 2020 will be included as outcomes. In this preliminary analysis, 324 744 deaths were observed among individuals aged 60 or older in the analytical period, irrespective of the year in which the death occurred. This substantial volume of mortality events supports the feasibility and statistical power of the analyses proposed in this protocol.

### Analysis plan

**Figure 2:**
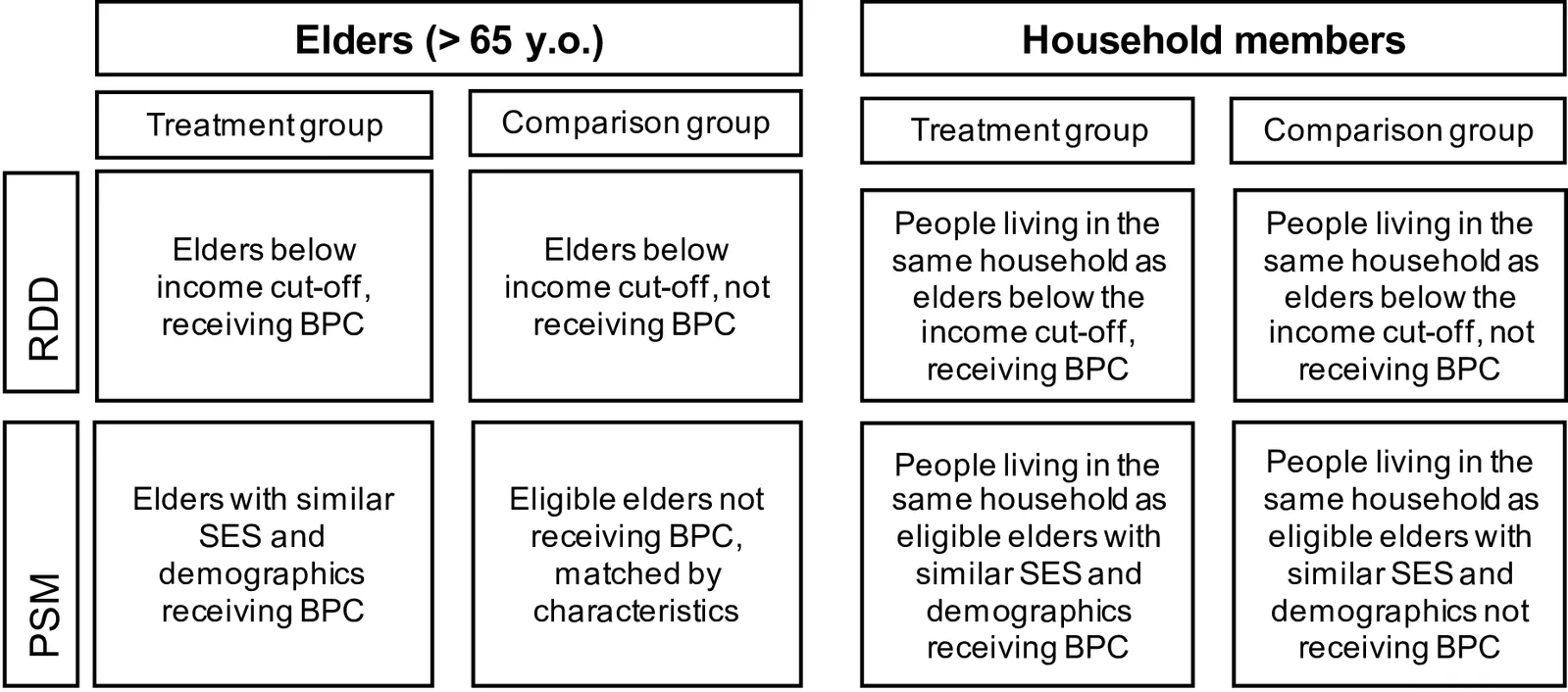
Summary of the methodological strategies to evaluate the impacts of the continuous cash benefit (BPC) on older people and household members. BPC, Benefício de Prestação Continuada; PSM, propensity score matching; RDD, regression discontinuity design; SES, Socioeconomic Status.

The BPC eligibility criteria provide an opportunity to employ quasi-experimental methods. We will triangulate across different methodological approaches to estimate the causal effects of the BPC on older people and their household members (see figure 2). We will use regression discontinuity design (RDD) and propensity score (PS)-based approaches. Each method addresses different sources of bias. While RDD leverages the programme’s age and income eligibility threshold to account for both observed and unobserved confounding around the cut-off, PS reduces confounding by balancing observable characteristics between treated and untreated individuals. Together, these methods strengthen causal inference by mitigating the risk of residual confounding and improving robustness across analytical strategies.

Discrepancies between RDD and inverse probability of treatment weighting (IPTW) estimates will be interpreted based on their distinct target populations. RDD estimates a local average treatment effect (LATE) for individuals near the eligibility thresholds, providing high internal validity against unmeasured confounding.^41 42^ In contrast, IPTW estimates a broader average effect under the assumption of conditional exchangeability.^43^ We will prioritise the consistency of findings across both methods, viewing RDD as the primary evidence for causal inference.

#### Regression discontinuity design

Regression discontinuity (RD) is a quasi-experimental design that uses a threshold of a continuous intervention assignment variable to distinguish individuals who receive the treatment from those who do not, thereby assessing the possible causal impact of the intervention.^41^ The underlying rationale is that individuals around the threshold are comparable in all observed and unobserved characteristics that may affect the outcome, differing only in their treatment with the intervention.^44^ The BPC programme uses clear income and age thresholds to separate the recipient from the non-recipient group. The validity of the RD design depends on one key assumption: continuity of potential outcomes and covariates at the cut-off. Although this assumption is not directly testable, it can be assessed through empirical checks, such as tests for discontinuities in baseline covariates and the density of the running variable around the threshold. ‘Continuity’ stipulates that the cut-off point must be exogenous, ensuring it is not coincidentally aligned with any other action occurring when units are assigned to the intervention group, and that, at the threshold, only the intervention assignment could have caused a discontinuity or jump in the outcome variable.^42 45^

At age 65, individuals may fail to satisfy the eligibility criteria for BPC benefits. Furthermore, even among those who are eligible, receipt of the BPC may be delayed or not occur due to limited awareness of eligibility, individual decision-making, regulatory changes and administrative barriers to access. Therefore, we implement a fuzzy RDD, as treatment assignment is imperfect: individuals below age 65 are always in the control group, while those aged 65 and above are not necessarily recipients of BPC. In a fuzzy design, treatment assignment at the cut-off serves as an instrumental variable for actual receipt of the BPC.^42^ Therefore, if discontinuities in the probability of treatment are observed, we will estimate the LATE. The first step will be to assess the discontinuity in the probability of receiving the BPC at the threshold across groups. This will be conducted separately using both age and income as assignment variables. Second, the assumption of non-manipulation for income will be tested visually and by using the manipulation test introduced by Cattaneo *et al*,^46^ building on the approach initially developed by McCrary.^47^ The test examines the distribution of observations around the cut-off point. A marked disparity in the density of observations immediately before and after the threshold would provide evidence suggesting manipulation. To ensure the validity of our RDD estimates, we will implement explicit decision rules based on diagnostic tests. The continuity assumption will be formally tested using the Cattaneo *et al*^46^ manipulation test, building on the McCrary^47^ density test, to detect any non-random sorting around the thresholds. If a statistically significant jump in observation density is found at the cut-off (p<0.05), the assumption of non-manipulation will be rejected, and the source of sorting will be investigated. In such cases, we will explore alternative bandwidths and rely on complementary causal approaches. Furthermore, we will test for discontinuities in predetermined baseline covariates; the RDD validity will be accepted only if these covariates are balanced across the threshold.^41^

Third, to evaluate the continuity assumption, we will (1) analyse the smoothness of the outcome variables in other thresholds of age and income (½ minimum wage or 60 years old) and (2) analyse the smoothness of baseline socioeconomic and demographic characteristics near the threshold. In this context, the treatment assignment probability should be the sole factor with a clearly distinguished difference in probability above and below the threshold.

If key assumptions are met, to assess the effect of the BPC on older adults, we will take advantage of the discontinuity in age (≥65 years old) and per capita income eligibility (ie, ¼ of minimum wage equivalent for each calendar year) as the assignment variable to estimate the causal effects of the BPC near the income eligibility threshold. When conducting the analysis using age as the assignment variable, we will restrict our analysis to people aged 60 or older with per capita income below ½ minimum wage. When conducting the analysis using income as the assignment variable, we will restrict the analysis to individuals aged 65 years or more and will test different per capita income bandwidths.

To address concerns regarding statistical power and time-varying policy environments, we will adopt a normalisation approach. This method stacks data from different periods (pre-2020 and post-2020), normalising the running variables (age and income) relative to their respective regulatory cutoffs. This pooling increases the number of observations near the thresholds, enhancing statistical precision and ensuring that the estimated LATE is representative of the entire study period despite regulatory shifts.^48 49^

We will use local polynomial regression, which focuses the analysis on observations just to the left and right of the cut-off, and tends to achieve better data fitting near the cut-off than global estimates.^50^ We will implement the robust bias-corrected bandwidth selection algorithm proposed by Calonico *et al*.^51^ Complementarily, for secondary outcomes with low event incidence, we will estimate RD effects using global regressions with a second-order polynomial, in order to improve statistical precision and stability while preserving identification at the cutoff.^52^ Finally, for mortality outcomes, we will also estimate treatment effects using time-to-event (ie, survival models) using the log of the proportional hazard model^53^ or the accelerated failure time.^54^

#### PS methods

We will additionally investigate the effects of BPC on the defined health outcomes using survival analysis following matching or IPTW.

Beneficiaries and non-beneficiaries will be paired or weighted to ensure comparable distributions of measured baseline characteristics.^43^ We will first restrict our analysis to individuals with per capita income up to one minimum wage, followed by a more focused restriction to those within ½ minimum wage (ie, the eligibility range for BPC). Second, the PS will be estimated using demographic and socioeconomic variables from the 100 Million Brazilian Cohort (eg, age, gender, race/ethnicity, educational level, housing conditions and geographic region). Two strategies may be applied:

1. Propensity score matching using kernel or nearest-neighbour algorithms with replacement; and
2. PS Weighting (IPTW) using the inverse probability of treatment to create a weighted pseudo-sample in which the distribution of observed covariates is balanced across beneficiaries and non-beneficiaries.

Both approaches aim to reduce observed confounding and to generate balanced groups for estimating the association between BPC receipt and mortality or survival outcomes. These matched or weighted groups will then be used to estimate the impact of BPC on mortality and survival outcomes.

#### Analysis of household members and subgroup analysis

Treatment and comparison groups for each analytical strategy are defined based on eligibility criteria and are detailed in online supplemental table S2 and material). To study the effects of BPC on the health outcomes of household members, we will use the same RDD or PS weighting analysis but attribute the eligibility and treatment variables for older people to household members. Individuals residing with eligible older adults receiving BPC benefits will be compared with those living in similar households where BPC benefits are not received. To account for the non-independence of observations within the same household, all analyses will adjust for intrahousehold clustering by using cluster-robust standard errors at the household level.

We will also examine whether the effects of the BPC vary across key demographic and socioeconomic subgroups—such as age, sex, race/ethnicity, key traditional population groups (ie, indigenous and quilombola communities), area deprivation and geographical region of Brazil.

#### Sensitivity analysis and other robustness checks

First, as recent changes in the legal eligibility criteria for the BPC have introduced important challenges for our identification strategy, particularly for the RDD, we will stratify analyses by period (ie, pre-2020, where the per capita eligibility income threshold was 1/4 of the minimum wage, vs post-2020, with a more flexible threshold of up to ½ of the minimum wage), in order to assess whether estimated effects are robust to changes in eligibility rules and to avoid conflating policy effects across distinct regulatory regimes. To further strengthen causal claims, we will implement placebo cut-off tests (falsification tests). We will artificially shift the age threshold (eg, to 60 and 70 years) and the income threshold (eg, to one minimum wage) where no policy change occurred. A finding of significant effects at these placebo points would indicate underlying bias, whereas a null result will reinforce that the observed effects at the 65 year/quarter-income cut-off are indeed causal.^40 55 56^

Second, to strengthen the validity of our findings, ensure data reliability and align with prior research on the BFP and its effects on mortality, we will restrict analyses to municipalities with high-quality mortality reporting, characterised by low levels of underreporting,^57^ thereby reducing measurement error in mortality outcomes and limiting bias due to differential data quality across municipalities.

Third, for individuals aged 65 and older, receipt of the BPC may overlap with eligibility for free public transportation benefits provided under the Statute of the Elderly.^58^ As these transportation benefits are implemented at the municipal level, such overlap could potentially lead to an overestimation of the independent effects of the BPC on older adults. Ideally, this concern would be addressed by identifying municipalities and implementation timing of free transportation policies, particularly in medium-sized and large-sized cities, and testing for heterogeneous effects accordingly. However, in the absence of harmonised data on municipal implementation, we will conduct robustness analyses stratified by municipality population size and poverty levels as indirect proxies for variation in local policy environments and implementation capacity. These analyses aim to assess whether estimated BPC effects are sensitive to contextual municipal characteristics.

Finally, we will investigate the interplay between the BPC and the BFP by examining the effects of the BPC on families that do or do not concurrently receive BFP benefits, to distinguish the independent effects of the BPC from potential complementarities or interactions between income-support programmes within the same households. These strategies follow recent recommendations on robustness and heterogeneity analyses in quasi-experimental evaluations.^59^

### Strengths and limitations

We have developed a detailed framework to evaluate the effects of BPC on health using the 100 Million Brazilian Cohort. We propose to triangulate findings from two analytical strategies that play distinct and complementary roles in our analytical strategy. The RDD offers a strong quasi-experimental framework by addressing both observed and unobserved confounding near the cut-off point that determines treatment assignment. While it relies on local comparisons, it provides policy-relevant estimates of marginal effects around the eligibility threshold. PS methods broaden the evaluation beyond the immediate vicinity of the threshold by balancing observed characteristics and improving comparability across groups. Rather than privileging a single approach, we will base our conclusions on the consistency of findings across methods and on the complementary insights that each approach provides. In addition, by estimating the effects of the BPC on morbidity and mortality in Brazil, our findings may inform debates on the role of unremarkable pensions within the social protection system, particularly regarding their potential health returns.

This study has important limitations that should be acknowledged. First, while the RDD provides a strong quasi-experimental framework under the continuity assumption, its estimates are restricted to individuals near the eligibility threshold and may not generalise to all beneficiaries. PS methods broaden external validity but rely on the assumption of unmeasured confounding. Also, potential selection bias may arise from the linkage process, as 14.2% of records from the BPC payment database remained unmatched due to inconsistent or missing NIS. While this likely reflects administrative data quality issues rather than systematic exclusion, we will conduct sensitivity analyses comparing the baseline characteristics of matched and unmatched individuals to evaluate if specific subgroups were disproportionately excluded. Regarding external validity, the RDD approach identifies a LATE, meaning the estimates are strictly valid for individuals near the eligibility thresholds (eg, age 65 and the specified income cutoffs). Consequently, results may not be directly generalisable to the broader elderly population with higher incomes or different age brackets. However, triangulation with IPTW methods aims to broaden external validity by estimating effects across a wider range of the low-income population, assuming conditional exchangeability.

Second, this study relies on the 100 Million Brazilian Cohort, which consists predominantly of low-income individuals. While this focus enhances internal validity for the most vulnerable groups, it may limit the generalisability of findings to the broader elderly population in Brazil. However, from a policy perspective, this population is the primary target of the BPC and the most dependent on the public health system (SUS), making these results highly relevant for social protection and health equity strategies.

Third, limitations inherent to the outcome data must be considered. Mortality data from SIM are generally of high completeness but may still be subject to regional variation in reporting quality. Hospitalisation data from SIH capture admissions within the public health system (SUS), responsible for over 70% of all hospitalisations,^37^ and therefore, do not include private-sector hospitalisations; however, given the socioeconomic profile of the cohort, reliance on SUS is expected to be higher than the average. Importantly, hospitalisations are a complex outcome. While reductions in avoidable admissions may reflect improved health status and disease management, increases in hospitalisation rates may indicate improved access to healthcare rather than worsening health. Moreover, from a health economics perspective, hospitalisations can also be interpreted as healthcare costs rather than direct measures of health status. We therefore interpret hospitalisation outcomes cautiously, distinguishing between preventable and non-preventable causes when possible and contextualising findings within broader patterns of healthcare access and utilisation.

Finally, the use of large-scale administrative databases from the 100 Million Brazilian Cohort provides substantial power due to its size (evidenced by over 324 000 observed deaths in the study period), which supports the feasibility of detecting marginal effects. To further enhance precision, we standardised the running variables (age and income) relative to their specific annual thresholds, which increases the number of observations near the eligibility thresholds by aggregating data across different regulatory periods. However, a limitation of this protocol is its focus on short-term effects, as the current available follow-up for BPC payment records linked to health outcomes is limited to a maximum of 4 years. Consequently, while the study offers high internal validity for immediate impacts, long-term inference regarding the cumulative effects of the pension on chronic disease progression or late-life mortality remains constrained. Future updates of the administrative linkage will be necessary to evaluate the programme’s sustainability and its long-term health returns.

### Expected results

By providing evidence on health-related outcomes beyond poverty reduction, our results can contribute to the design and evaluation of income-support policies targeting older adults and other vulnerable populations. Importantly, because the BPC differs from CCT programmes such as Bolsa Família in that it does not require compliance with health or education conditionalities, any observed impacts are likely to operate primarily through income security and reduced socioeconomic vulnerability rather than through conditionalities. Finally, the findings from the studies are likely to support evidence-based adjustments to the programme, such as benefit adequacy, eligibility thresholds and coordination with health and long-term care services. In particular, the LATEs estimated around the eligibility thresholds are directly informative for assessing the likely impact of marginal policy changes.

## Supplementary material

10.1136/bmjopen-2026-119914 online supplemental file 1
