# Supplementary material for: Impact of a non-contributory pension for older adults on hospitalisations and mortality: a protocol for a natural experimental study of the Brazilian continuous cash benefit (BPC)
Source: BMJ Open. 2026 Jul 3;16(7):e119914. doi: 10.1136/bmjopen-2026-119914 (PMC13343044; doi:10.1136/bmjopen-2026-119914)
Supplement: online supplemental file 1 [file bmjopen-16-7-s001.docx]

**Supplementary materials**

**Table S1 (Supplementary): Template for Intervention Description and Replication (TIDieR)**

| **TIDieR Item** | **Program Description** |
| --- | --- |
| **1. Name** | Benefício de Prestação Continuada (BPC) |
| **2. Why** | **Provides non-contributory income support to older adults and persons with disabilities in socioeconomic vulnerability, aiming to reduce poverty, inequality, and social exclusion.** |
| **3. What (materials)** | **Eligibility – Older adults (65+ years): - Aged 65 years or older. - No prior contributions to INSS required. - Household per capita income below the legally defined threshold. - Not receiving other contributory INSS benefits. - Registered in CadÚnico.  Household members: - Individuals of any age co-residing with an eligible older BPC beneficiary. - Identified through household composition data in CadÚnico. - Not direct beneficiaries, but potentially affected through household income sharing.** |
| **4. What (procedures)** | Established under Article 203 of the 1988 Federal Constitution and regulated by the Organic Law of Social Assistance (LOAS, 1993). |
| **5. Who provided** | Managed by the Federal Government, coordinated by the Ministry of Social Development and operationalised by the National Institute of Social Security (INSS). Financed by the National Social Assistance Fund. |
| **6. How** | Non-contributory social assistance benefit. Does not include 13th salary or survivor pension. Eligibility for persons with disabilities requires medical and social assessments by National Institute of Social Security (INSS). |
| **7. Where** | Monthly payments deposited into beneficiary-designated bank accounts or accessed via magnetic cards. |
| **8. When and how often** | Monthly payments starting from the approval date of the application, following the official National Institute of Social Security (INSS) payment calendar. |
| **9. How adherence was monitored** | The National Institute of Social Security (INSS) verifies income eligibility using the Unified Registry for Social Programs (CadÚnico) and conducts medical and social evaluations for persons with disabilities. |
| **10. Modifications** | Eligibility criteria, procedures, and benefit amounts changed over time.  Main variations included:  2003: Eligibility age reduced from 67 to 65 years. 2007: Income threshold formalised and medical assessment required. 2009: Adoption of WHO ICF for disability assessment. 2018: CadÚnico registration became mandatory. 2021: Flexible income thresholds and revised evaluation rules introduced. |
| **11. How the intervention was provided** | Implemented nationwide according to federal regulations and administered locally through National Institute of Social Security (INSS) service networks. |
| **12. Comments** | Changes in vulnerability status or income may result in suspension of benefits, requiring reassessment for reinstatement. |

**Table S2: Criteria for Selection of Treatment and Comparison Groups in the BPC Study**

| **Group** | **Eligibility Criteria**^1^ | **Treatment Group Selection** | **Comparison Group Selection** | **Follow-up** |
| --- | --- | --- | --- | --- |
| **Elders (RDD)** | Age ≥ 65 years below the income threshold of ¼ of the minimum wage (MW) | (i) Elders (≥65 years) below the income threshold ;  (ii) Elders (≥65 years) below the income threshold | 1. Elders (≥65 years) above the income threshold 2. Individuals aged 60-64 below the income threshold | Any length of time |
| **Elders (PSM)** | Age ≥ 65 years; Various socioeconomic (SES) and demographic variables | Elders (≥65 years) who receive BPC, weighted using IPTW (inverse propensity treatment weights) using socioeconomic and demographic variables | Elders (≥65 years) who do not receive BPC, matched **o**r weighted on SES and demographic variables | Any length of time |
| **Household Members (RDD)** | Household members living with elderswhose income below ¼ of MW | Members living with:  (i) Elders (≥65 years) below the income threshold ;  (ii) Elders (≥65 years) below the income threshold | Members living with elders who:   1. Elders (≥65 years) above the income threshold   (ii) Individuals aged 60-64 below the income threshold | Any length of time |
| **Household Members (PSM)** | Household members living with elders  Various SES and demographic variables | Members living with elders, matched **o**r weighted using PSM on SES and demographic variables | Members living with elders who do not receive BPC, matched **o**r weighted on SES and demographic variables | Any length of time |

^1^ Income is measured as per capita monthly income.
