# Supplementary material for: Impact of a non-contributory pension for older adults on hospitalisations and mortality: a protocol for a natural experimental study of the Brazilian continuous cash benefit (BPC)
Source: BMJ Open. 2026 Jul 3;16(7):e119914. doi: 10.1136/bmjopen-2026-119914 (PMC13343044; doi:10.1136/bmjopen-2026-119914)
Supplement: Supplementary file 2 [file bmjopen-2026-119914.reviewer_comments.pdf]

PEER REVIEW HISTORY

BMJ Open publishes all reviews undertaken for accepted manuscripts. Reviewers are asked to complete a checklist review form and are provided with free text boxes to elaborate on their assessment. These free text comments are reproduced below.

ARTICLE DETAILS

Title (Provisional)

Impact of a Non-Contributory Pension for Older Adults on Hospitalizations and Mortality: A Protocol for a Natural Experimental Study of the Brazilian Continuous Cash Benefit (BPC -Elderly)

Authors

Silva, Samuel Araujo Gomes da; Dias, Francine de Souza; Guimarães, Joanna MN; Casais, Gustavo; Moncayo, Ana L.; Ruiz, Ronal Alejandro Grijalva; Machado, Daiane Borges; Alves, Flávia Jôsi Oliveira; Katikireddi, Srinivasa Vittal; Leyland, Alastair H.; Barreto, Maurício Lima; Ribeiro , Rita de Cássia; Craig , Peter; Pescarini , Julia

---

VERSION 1 - REVIEW

**Reviewer**	**1**
**Name**	**Kumar, Naresh**
**Affiliation**	**ITS Engineering College, Applied Sciences and Humanities**
**Date**	**05-May-2026**
**COI**	**None**

---

1. The authors should more clearly highlight how this protocol advances beyond prior evaluations, particularly those cited in the introduction.

2. The authors should clarify specifically how discrepancies between the two methods will be interpreted.

3. The authors should clarify how this heterogeneity will be handled analytically to avoid bias in treatment assignment.

4. The authors should discuss the potential for selection bias and how it may affect external validity.

5. The manuscript acknowledges that hospitalization can reflect both improved access and worsening health. However, the analytical strategy for disentangling these competing mechanisms is not sufficiently detailed. The authors should clarify how they will interpret conflicting trends.

6. The authors should discuss implications for statistical power and long-term inference.

7. While the manuscript proposes several tests (e.g., McCrary test, covariate balance), more detail is needed on thresholds for validity and how violations will be addressed. The authors should explicitly state decision rules for accepting or rejecting RDD assumptions.

8. The authors should provide a more comprehensive strategy to control for time-varying policy environments.

9. The reliance on a low-income cohort (100 Million Brazilian Cohort) enhances internal validity but limits generalizability to the broader elderly population in Brazil. This limitation should be more explicitly discussed, especially in the context of policy implications.

VERSION 1 - AUTHOR RESPONSE

Reviewer 1's Comments

1. **Highlighting advances beyond prior evaluations (The authors should more clearly highlight how this protocol advances beyond prior evaluations, particularly those cited in the introduction):**

The Introduction now explicitly states how this study advances beyond prior correlational research by leveraging the 100 Million Brazilian Cohort and applying a natural experiment design (Fuzzy RDD) to mitigate unmeasured confounding at the individual level, which was a gap in previous evaluations (see pages 3 and 4).

2. **Interpretation of RDD vs. IPTW discrepancies (The authors should clarify specifically how discrepancies between the two methods will be interpreted):**

We clarified in the "Analysis plan" that RDD estimates a Local Average Treatment Effect (LATE) near the thresholds, while IPTW estimates effects across a wider range of the population. We will base our conclusions on the consistency of findings across methods and the complementary insights each provides. RDD results will be prioritized for internal validity (see pages 12 and 13).

3. **Handling heterogeneity and time-varying policy (The authors should clarify how this heterogeneity will be handled analytically to avoid bias in treatment assignment):**

We implemented a strategy to address heterogeneity in treatment assignment arising from changes in eligibility rules over time. Specifically, we normalize the running variables (age and income) relative to their annual regulatory thresholds and adopt a RDD framework across regulatory periods. In addition, as described in the sensitivity analyses, we stratify

analyses by policy period (pre- and post-2020) to assess whether estimates are robust to changes in eligibility criteria. Together, these approaches reduce the risk of bias arising from time-varying treatment assignment mechanisms (see pages 14 and 15).

4. **Selection bias and external validity (The authors should discuss the potential for selection bias and how it may affect external validit).**

- Selection Bias: We acknowledge that 14.2% of BPC records were unlinked due to NIS inconsistencies. We added a plan for sensitivity analysis to compare baseline characteristics between linked and unlinked individuals to check for systematic exclusion (see pages 18 and 19).
- External Validity: We have standardized the text to use "Treated/Untreated" to clearly define the intervention groups. We discuss that while the cohort is low-income, it represents the primary target of the BPC and the most SUS-dependent population, making results highly relevant for policy (see page 19).

5. **Disentangling hospitalization mechanisms (The manuscript acknowledges that hospitalization can reflect both improved access and worsening health. However, the analytical strategy for disentangling these competing mechanisms is not sufficiently detailed. The authors should clarify how they will interpret conflicting trends):**

We now state that changes in hospitalization rates will be interpreted cautiously. We distinguish between avoidable admissions (which should decrease with better health management) and potential increases in admissions due to improved access to care (e.g., better ability to afford transportation to the hospital). Reductions in primary care-sensitive conditions will be interpreted as improved health management (see pages 11 and 12).

6. **Statistical power and long-term inference (The authors should discuss implications for statistical power and long-term inference).**

We have addressed this point by clarifying our strategies for ensuring statistical precision and acknowledging the timeframe of our causal estimates: 1) Statistical Power: We have highlighted that the large sample size of the 100 Million Brazilian Cohort (with over 324,000 mortality events already identified) provides sufficient power for cause-specific analyses. Additionally, we have now explicitly standardized the running variables (age and income) relative to their specific annual thresholds. This procedure allows us to pool observations across regulatory regimes, increasing the volume of data near the eligibility cutoffs and significantly enhancing statistical precision for cause-specific analyses; 2) Long-term Inference: We have expanded the 'Strengths and limitations' section to discuss that our current

follow-up (maximum 4 years) primarily captures short-term to medium-term effects. We acknowledge that while this provides critical evidence on immediate health impacts, long-term inference is limited by the current data window, and future longitudinal linkages will be required to assess late-life health trajectories (see pages 12-15).

7. **Decision rules for RDD assumptions (While the manuscript proposes several tests (e.g., McCrary test, covariate balance), more detail is needed on thresholds for validity and how violations will be addressed. The authors should explicitly state decision rules for accepting or rejecting RDD assumptions):**

We have now explicitly defined our decision rules for RDD validity. Specifically, we will reject the non-manipulation assumption if the Cattaneo et al. density test yields $P < 0.05$. Additionally, following the methodological rigor of recent studies in JAMA (Pomirchy et al., 2025) and Nature (Eyting et al., 2025), we have added placebo cutoff tests at age 60/70 and 1 minimum wage as falsification checks to ensure the BPC eligibility is the sole driver of the observed health discontinuities (see page 14).

8. **To control for time-varying policy environments (The authors should provide a more comprehensive strategy to control for time-varying policy environments).**

We have implemented a multi-layered strategy to strictly control for time-varying policy environments and regulatory shifts: 1) Temporal Stratification: As detailed in the 'Sensitivity analysis' section, we stratify all analyses by regulatory period. This prevents the conflation of effects from the original 1/4 minimum wage threshold with the more flexible criteria introduced in 2021; 2) Threshold normalization: We adopted an approach that normalizes the running variables (age and income) relative to their specific regulatory cutoffs in different years. This ensures that the estimated LATE is not biased by the shift in thresholds over time; 3) Control for Municipal Policy Heterogeneity: To account for local variations (e.g., free public transportation for the elderly, which varies by city), we conduct robustness analyses stratified by municipality size and poverty levels as proxies for local implementation capacity; 4) Program Interplay: We explicitly investigate the interaction between the BPC and the Bolsa Família Programme (BFP) to disentangle the independent effect of the pension from other cash transfer dynamics within the household (see pages 14–15 and 16–17).

9. **Generalizability of the low-income cohort (The reliance on a low-income cohort (100 Million Brazilian Cohort) enhances internal validity but limits generalizability to the broader elderly population in Brazil. This limitation**

should be more explicitly discussed, especially in the context of policy implications):

We expanded the "Strengths and limitations" section to discuss that while the cohort is low-income, it represents the primary target of the BPC and the most SUS-dependent population, making the results highly policy-relevant (see pages 18 and 19).

Updated References used in this Revision:

- Pomirchy M, et al. Herpes Zoster Vaccination and Dementia Occurrence. JAMA. 2025;333(23):2083-2092.
- Eyting M, et al. A natural experiment on the effect of herpes zoster vaccination on dementia. Nature. 2025.
- Bermingham C, et al. Estimating the Effectiveness of First Dose of COVID-19 Vaccine Against Mortality in England: A Quasi-Experimental Study. Am J Epidemiol. 2022.
- Cattaneo MD, et al. A Practical Introduction to Regression Discontinuity Designs: Extensions. Cambridge University Press; 2024.
- Tang Y, et al. Statistical power for the comparative regression discontinuity design. Psychol Methods. 2018.

Table 3 has been updated to incorporate a broader range of ICD-10 chapters (including Chapters V, VI, XIV, and XIX), following the availability of additional cause-of-hospitalization data in the linked SIH database, which allows for a more comprehensive evaluation of the BPC's impact on morbidity patterns.

We believe these revisions fully address the concerns raised and look forward to your decision.
